# Diacylglycerol-evoked activation of PKC and PKD isoforms in regulation of glucose and lipid metabolism: a review

**DOI:** 10.1186/s12944-020-01286-8

**Published:** 2020-05-28

**Authors:** Katarzyna Kolczynska, Angel Loza-Valdes, Izabela Hawro, Grzegorz Sumara

**Affiliations:** Nencki Institute of Experimental Biology, Polish Academy of Sciences, 3 Pasteur Street, 02-093 Warszawa, Poland

**Keywords:** Diacylglycerol (DAG), PKC, PKD, Metabolism, Insulin signaling

## Abstract

Protein kinase C (PKC) and Protein kinase D (PKD) isoforms can sense diacylglycerol (DAG) generated in the different cellular compartments in various physiological processes. DAG accumulates in multiple organs of the obese subjects, which leads to the disruption of metabolic homeostasis and the development of diabetes as well as associated diseases. Multiple studies proved that aberrant activation of PKCs and PKDs contributes to the development of metabolic diseases. DAG-sensing PKC and PKD isoforms play a crucial role in the regulation of metabolic homeostasis and therefore might serve as targets for the treatment of metabolic disorders such as obesity and diabetes.

## Diacylglycerol (DAG) – the structure and sources

Diacylglycerol (DAG) is a neutral lipid involved in various metabolic pathways in the cell. It is an important component of membranes that also acts as a secondary messenger. It was shown that DAG is involved among others in multiple processes and pathways, e.g. protein transport, vesicle secretion, insulin signaling, cell growth, and proliferation [1–3]. In the cell, DAG can be either synthesized during de novo lipid biosynthesis or generated from other intracellular lipid species. Different reactions contributing to the generation of DAG occur in various subcellular compartments like plasma membrane, Golgi network, endoplasmic reticulum (ER), and lipid droplets. During de novo biosynthesis of triacylglycerol (TAG) and phospholipid (PL), DAG is produced as an intermediate by acyltransferases and phosphohydrolases [4, 5]. DAG is also generated from TAG stored in either cytoplasmic and ER-associated lipid droplets in a reaction catalyzed by lipases or in the plasma membrane and Golgi complex from PL by acyltransferases [6].

**Fig. 1:**
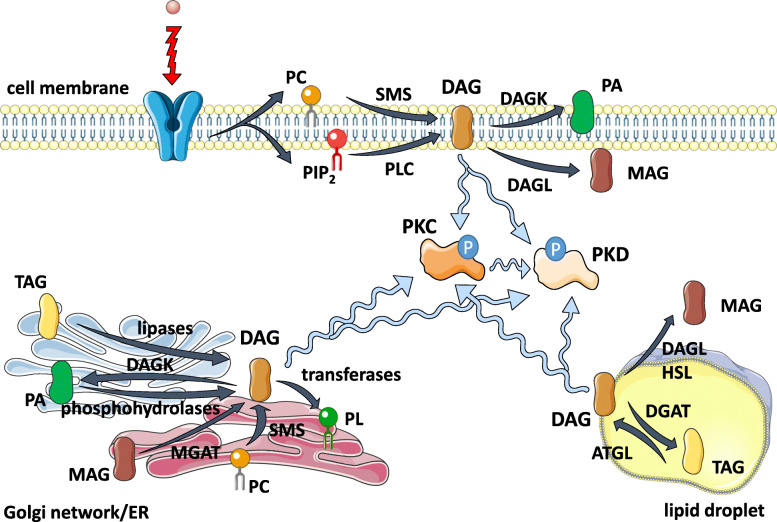
Sources of diacylglycerols (DAG) in the cell. Intracellularly, DAG is produced as an intermediate during de novo biosynthesis of triacylglycerol (TAG) and phospholipid (PL), and during catabolism of TAG stored in either cytoplasmic and endoplasmic reticulum-associated lipid droplets or PL in the plasma membrane and Golgi complex. Generated DAG locally recruits and promotes activation of conventional and novel protein kinases C (PKCs) and protein kinases D (PKDs). For detailed description see the text. ATGL – adipose triglyceride lipase, DAGK – diacylglycerol kinase, DAGL – diacylglycerol lipase, DGAT – diglyceride acyltransferase, ER – endoplasmic reticulum, HSL – hormone-sensitive lipase, MAG – monoacylglycerol, MGAT – monoacylglycerol-O-acyltransferase, PA – phosphatidic acid, PC – phosphatidylcholine, PIP_2_ – phosphatidylinositol 4,5-bisphosphate, PLC – phospholipase, SMS – sphingomyelin synthase

**Fig. 2:**
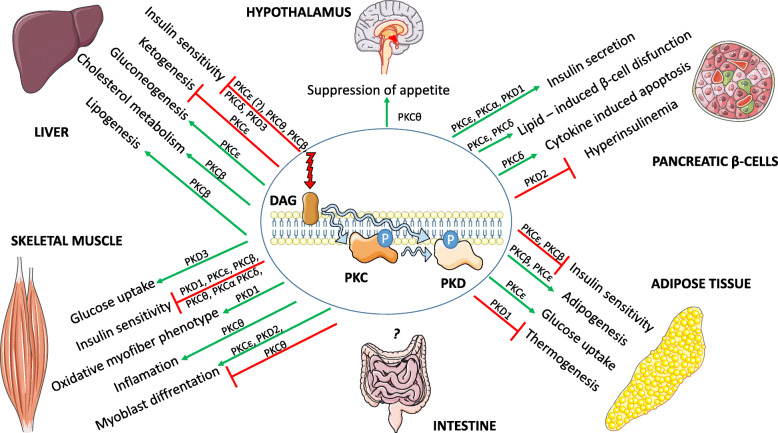
The role of PKC and PKD isoforms in various tissues and organs

DAG consists of two fatty acids chains covalently bound to glycerol through ester bonds. There are three different isomeric forms of DAG: sn-1,2, sn-2,3 and rac-1,3 DAG [7]. It was shown that different enzymes could discriminate between DAG isomers [3, 8]. Moreover, proteins interacting with DAG, depending on their function, are localized in different subcellular compartments. Thus, it was suggested that DAG stereo/regioisomers are localized in distinct parts of the cell and play different roles in cell signaling [6, 7]. DAG can be generated from TAG during the first step of lipolysis. During this process, TAG is hydrolyzed into fatty acid and DAG by TAG lipases [6]. TAG stored in cytoplasmic and ER-associated lipid droplets is accessible to different kinds of lipases. Adipose triglyceride lipase (ATGL) is the primary enzyme responsible for the hydrolysis of TAG localized in the cytoplasm. It generates rac-1,3 DAG and is the only known lipase that can hydrolyze TAG at the sn-2 position. Moreover, in the presence of its activator - comparative gene identification-58, ATGL can also hydrolyze TAG to sn-2,3 DAG [3]. Another enzyme that can hydrolyze cytoplasmic TAG is hormone-sensitive lipase (HSL), which produces sn-2,3 DAG. At the same time, DAG generated on cytoplasmic lipid droplets is a substrate for HSL and diacylglycerol lipase β (DAGLβ), which hydrolase it to fatty acid and monoacylglycerol (MAG). It was shown that HSL specifically hydrolyzes rac-1,3 DAG and sn-2,3 DAG and shows a preference for polyunsaturated fatty acids [8, 9]. Cytoplasmic DAG can be also re-esterified to TAG via diglyceride acyltransferase (DGAT2). At ER and Golgi network, DAG is generated as mentioned above during TAG hydrolysis, but also through de novo biosynthesis from MAG or phosphatidic acid (PA) in reactions catalyzed by monoacylglycerol-*O*-acyltransferase (MGAT) and PA phosphohydrolase respectively or as a side product during sphingomyelin synthesis. The main isomeric form present at ER and Golgi network is sn-1,2 DAG, which can be metabolized by DGAT1 and DGAT2 to TAG, diacylglycerol kinase (DAGK) to PA or enzymes involved in PL formation [3, 10, 11]. At the plasma membrane, sn-1,2 DAG is generated during sphingomyelin synthesis or cleavage of phosphoglycerol from glycerophospholipids in a reaction catalyzed by phospholipase C (PLC) [3, 12]. Phosphatidylinositol-specific PLC (PI-PLC) hydrolyzes phosphatidylinositol 4,5-bisphosphate (PIP_2_) and generates 1,2-sn DAG, which specifically activates protein kinase C and subsequently protein kinase D [2, 3, 13].

DAG is not only generated intracellularly but is also an intermediate of extracellular lipid metabolism and can be supplied with diet. Extracellular DAG is a product of TAG hydrolysis during digestion and in the catabolism of lipoprotein-associated TAG in the bloodstream. Since DAG generated in the digestive system or in circulation is usually immediately hydrolyzed to MAG and fatty acid, it is probably not involved in the regulation of signaling pathways.

Intracellular changes in DAG level are affecting various signaling pathways and processes. For instance, an elevated level of DAG in many tissues is correlated with impaired cell metabolism and pathogenesis of metabolic disorders like insulin resistance. Additionally, a high-fat diet (HFD) feeding in animal models leads to the development of metabolic disorders accompanied by an increased level of DAG in peripheral tissues [14]. ATGL-deficiency in mice that leads to decreased DAG level and increased TAG content, protects from HFD-induced insulin resistance and glucose intolerance [15]. This finding supports the statement that DAG can disrupt insulin signaling in peripheral tissues. The described phenomenon is caused by DAG acting as a secondary messenger through interactions with several proteins in the cell. It was shown that DAG can interact with proteins containing the C1 domain that represents the recognition motif for DAG and phorbol esters. C1 domain was found among others in protein kinases C (PKCs), protein kinases D (PKDs), DAGKs, Rac GTPase-activating proteins (Rac-GAPs), Ras guanyl nucleotide-releasing proteins (RasGRPs), and mammalian uncoordinated-13 proteins (Munc13s) [16–20]. Different proteins within the families containing the C1 domain differ in affinity to DAG and phorbol esters. However, DAG is involved in the regulation of various signaling pathways through interaction with these proteins. Due to a large amount of information available up to date about different classes of DAG-sensing proteins involved in regulation of various physiological processes, this review is focused only on DAG-dependent PKCs and PKDs kinases which DAG directly activates, and their function in the regulation of lipid and glucose metabolism in various tissues.

## PKC isoforms – structure and classification

PKCs are a family of serine/threonine kinases involved in various processes in cells including proliferation, differentiation, cell survival, and apoptosis [21, 22]. Moreover, PKC plays a vital role in the pathogenesis of such diseases like diabetes or cancer [23, 24]. PKC family is composed of three different subgroups: conventional (cPKC), the novel (nPKC) and atypical (aPKC). PKCα, β1, β2, and γ belong to cPKC, PKCδ, ε, η and θ are nPKC, whereas aPKC comprises of PKCζ and λ/ι. All PKCs consist of the N-terminal regulatory region and C-terminal catalytic region (kinase domain) [1]. The regulatory region of all PKCs contains an autoinhibitory pseudosubstrate segment. Binding of secondary messengers or proteins scaffolds to specific modules in the regulatory region controls the position of the pseudosubstrate segment in or out of the substrate-binding site. Therefore it regulates the activity of the PKCs [1]. All of the PKCs contain at least one C1 domain with different affinity for DAG. Both cPKC and nPKC contain two C1 domains: C1A and C1B. However, it was shown that the C1B in nPKC has a much higher affinity to DAG than C1B in cPKC [25]. In both subgroups, C1B is considered as a primary DAG sensor [26]. Thus, nPKC respond to an increase in DAG content alone, whereas cPKC needs additional increase in intracellular Ca^2+^ to be fully activated [25]. The C1 domain of aPKC has no affinity to DAG and acts as a part of the autoinhibitory segment [27]. cPKC and nPKC are activated specifically via 1,2-sn DAG, usually generated by PLC [7]. cPKC also contains Ca^2+^-sensing C2 domain, which binds PIP_2_ in the plasma membrane. Similarly, C2 domain is present in nPKC. However, this domain cannot sense Ca^2+^ and bind PIP_2_ [1]. aPKC contains the PB1 domain, which mediates binding to protein scaffolds [27].

PKCs are physiologically activated by various extracellular signals transduced by hormones, growth factors, cytokines or antigens [28]. cPKC and nPKC respond to the elevation of intracellular DAG, which binds to PKCs and causes their activation and translocation to the membranes. Ca^2+^-dependent signals lead to rapid activation of cPKC, whereas nPKC may also be activated at the Golgi network and other cellular membranes in a more sustainable manner [29]. The presence of activated PKCs on internal membranes leads to the phosphorylation of various interacting proteins. For instance in vitro studies and experiments performed in cells expressing insulin receptor showed that PKCs phosphorylate specific serine/threonine residues of insulin receptor and its intracellular substrates belonging to the family of insulin receptor substrates (IRSs), causing impairment of insulin signaling pathway [30–35]. Abundance and role in metabolism regulation of different members of the PKC family differ depending on the type of tissue and will be further discussed below.

## PKDs – downstream effectors of PKCs and DAG

Similarly to PKCs, PKDs are DAG-activated family of serine/threonine kinases involved in various processes and pathways in the cell. However, substrate specificity varies between PKCs and PKDs and they play different roles in cell signaling. PKDs are calmodulin-dependent kinases and they regulate such processes as vesicle trafficking, cell differentiation, motility and apoptosis [36–38]. PKD family is composed of three isoforms: PKD1, PKD2, and PKD3. All PKDs consist of N-terminal regulatory domains (C1 domains and autoinhibitory PH domain) and the C-terminal catalytic region (kinase domain). PKDs contain two DAG-binding C1 domains: C1a and C1b, but DAG preferably binds to the C1a domain [39]. At N-terminus PKD1 and PKD2, but not PKD3 contain additionally hydrophobic Ala(/Pro)-rich region, which potentially can be inserted into the membranes [40]. Moreover, at C-terminus PKD1 and PKD2 contain a PDZ-binding motif with an autophosphorylation site [41].

PKDs are activated by extracellular signals transduced by hormones, growth factors, cytokines, and neurotransmitters [38]. DAG in cell membranes recruits PKD through its C1 domains and induces conformational changes that abrogate PKDs autoinhibitory mechanism, leads to autophosphorylation at C-terminus of PKD1 and PKD2, and subsequently activates PKDs [2, 42]. Next, DAG-activated PKC phosphorylates serine residues in the PKDs activation loop located in the kinase domain, which in turn causes further conformational changes resulting in the maximal increase of catalytic activity of PKDs [38]. PKC phosphorylates Ser738 and Ser742, Ser706 and Ser710, Ser731 and Ser735 in human PKD1, PKD2 and PKD3, respectively [2]. Both cPKC and nPKC can activate PKDs, however, it was shown that PKDs are phosphorylated mainly by nPKC [43, 44]. Prolonged presence of extracellular stimuli leads to autophosphorylation of a serine residue in the PKDs activation loop, therefore to PKC-independent activation of PKDs and induction of long-term effects of PKD on cell signaling [45, 46]. PKDs can be found in different cell compartments, including Golgi and plasma membranes, nucleus, cytoplasm, and mitochondria. Their activity depends on the availability of local DAG. PKDs are widely expressed in mammals but their level and specific role in cell signaling can vary between tissues, which will be further discussed below.

## DAG-evoked activation of PKCs and PKDs suppresses insulin signaling in hepatocytes

Liver plays a central role in metabolic homeostasis by regulating glucose, lipid and protein metabolism. Under physiological conditions such as feeding, hepatic insulin stimulation promotes glucose uptake, lipogenesis and inhibits gluconeogenesis. Conversely, upon fasting, glucagon stimulates glycogenolysis, gluconeogenesis and inhibits lipogenesis. Hepatic response to insulin and glucagon is dysregulated in subjects with insulin resistance and type 2 diabetes [47]. Of note, a plethora of preclinical data and human studies suggests that abnormal accumulation of lipidic molecules and their byproducts such as triglycerides, ceramides, DAGs and long-chain fatty CoA molecules are mechanistically linked to the development of insulin resistance and non-alcoholic fatty liver disease (NAFLD) [48–50]. At the molecular level, lipid sensing in the liver requires a complex regulatory network. As mentioned above; this review describes a DAG-sensitive PKC and PKD isoforms and their role in the regulation of metabolism.

The PKC family is the most studied in the context of hepatic metabolism. PKCε was proposed as a DAG sensing kinase functionally linked to the insulin signaling in the liver. It was shown that the accumulation of sn-1,2-DAG activates PKCε. However, exact mechanisms leading PKCε activation in the liver are still under debate [51, 52]. Upon activation, PKCε induces phosphorylation of insulin receptor substrate 1 (IRS1) at Ser1101, which blocks insulin signaling. A number of studies using genetic approaches to suppress PKCε signaling by using antisense oligonucleotides or by the targeted deletion in the whole body of mice have shown that inactivation of PKCε protects against insulin resistance induced by short and long term high-fat diet feeding [49, 53, 54]. Nevertheless, Brandon and colleagues have shown that liver-specific inactivation of PKCε does not affect insulin signaling in this organ. By contrast, the specific inactivation of PKCε in the adipose tissue might be involved in crosstalk with the liver, which elicits changes at hepatic gene expression level affecting metabolic fitness in this organ [55]. In addition, further research has suggested that PKCε deletion in the liver promotes ketogenesis and paradoxically suppresses gluconeogenesis [56]. Therefore, the relevance of PKCε in hepatic insulin signaling must be interpreted cautiously. Although there is plenty of evidence that suggests a clear biological role of different lipid species in the development of insulin resistance and NAFLD, PKCε signaling in this context requires further investigation [57].

Despite the fact that PKCε is the best characterized PKC isoform in the liver, other isoforms also seem to play a role in the regulation of hepatic metabolism. PKCθ is highly induced in the hepatic cell line, HepG2, upon insulin and glucose stimulation, which correlates with the degradation of the IRS-1 leading to impaired insulin signaling. Moreover, genetic inactivation of PKCθ with siRNA ameliorates insulin resistance in cells [58]. Additionally, PKCθ is activated by Ca^2+^ signaling upon hypoxic stress in hepatic stellate cells, triggering autophagy [59]. However, the physiological contribution of PKCθ in vivo is still controversial as deletion of PKCθ in whole body of mice leads to higher susceptibility to develop obesity, insulin resistance and lower energy expenditure [60]. Therefore, to clearly delineate the function of PKCθ in the regulation of hepatic metabolism, the generation of animals carrying a liver-specific deletion of PKCθ would be required.

The novel PKCδ has also been linked to the onset of insulin resistance. Acute elevation of free fatty acids (FFAs) by an intra-venus infusion of lipids and heparin activates the axis of PKCδ – NADPH oxidase increasing oxidative stress which suppresses insulin signaling [61, 62]. In line with these findings, it has been proposed that PKCδ deletion in hepatocytes upregulates the antioxidant system in the liver [63]. Consistently, deletion of PKCδ in the whole body of mice revealed a reduced expression of key pro-apoptotic genes and caspase 9 in a model of nonalcoholic steatohepatitis induced by a choline-deficient diet (MCD). However, the authors did not find significant differences in pro-fibrotic genes expression after 8 weeks of MCD diet in PKCδ-deficient mice compared to the control littermates [64]. In the genetic models of obesity, mice carrying inactivation mutation in the leptin receptor (so-called *db/db* mice) and in Zucker rats, PKCδ deficiency prevents hepatic triglyceride accumulation [65, 66], promotes insulin signaling by restoring AKT as well as glycogen synthases kinase β (GSK3β) activity and induces glucose uptake [65, 67]. Moreover, increased genetic susceptibility to develop diabetes in C57BL/6 J compared to S129S6/Sv strain of mice [68] could be partially related to changes in the locus activity of the gene encoding PKCδ [69]. In line with these findings, liver-specific overexpression of PKCδ aggravates diabetes and promotes hepatosteatosis. Moreover, hepatic mRNA levels of PKCδ correlate positively with fasting glucose and circulating triglycerides in obese people [69].

Another essential hallmark in the onset of fatty liver disease is dysregulation of cholesterol metabolism. PKCβ is a major regulator of cholesterol and fatty acid metabolism in the liver [70]. High-fat diet and high cholesterol diets increase PKCβ expression [71, 72]. Mechanistically, PKCβ phosphorylates and controls the nuclear translocation of Farnesoid X receptor (FXR) a master regulator for cholesterol removal through its conversion into bile acids in the liver [73]. Conversely, full inactivation of PKCβ in mice promotes diet-induced gallstone disease [74]. On the other hand, PKCβ-deficient mice are protected against diet-induced obesity, insulin resistance and ectopic accumulation of fat in the liver [75]. Furthermore, it has been shown that PKCβ activation, both in vitro and in vivo with insulin sustains de novo lipogenesis by regulating the expression and activity of sterol regulatory element-binding protein 1c (SREBP-1c) an essential regulator of the lipogenic machinery [76].

PKD isoforms represent another group of DAG sensing kinases in hepatocytes. Moreover, PKC isoforms can activate PKDs in multiple cell types [77]. Recently, it was shown that out of three PKD isoforms described (PKD1, PKD2, PKD3) only PKD3 is significantly expressed in hepatocytes. PKD3 can be activated in hepatocytes in response to the elevation in DAG content in the hepatocytes and livers of mice fed HFD [14]. Moreover, it was demonstrated that PKD3 suppresses insulin signaling in mice fed HFD, but the exact molecular mechanisms need to be identified. Hence, mice with liver-specific deletion of PKD3 present better insulin sensitivity and consequently glucose handling [14]. However, activation of insulin signaling in mice deficient for PKD3 is associated with enhanced lipogenesis and consequently leads to the accumulation of triglycerides and cholesterol in the liver [14]. Interestingly, the deletion of PKD3 in immune cells promotes liver fibrosis by activating the production of transforming growth factor β (TGFβ), a classical pro-fibrotic cytokine, by hepatic macrophage [78]. On the other hand, the deletion of PKD1 specifically in adipocytes protects the development of od liver steatosis evoked by HFD feeding [79].

In conclusion, a number of PKC and PKD isoforms in the liver present a spectrum of non-redundant functions in the regulation of hepatic metabolism. Generally, PKCs and PKD3 block insulin signaling at different levels contributing to the development of insulin resistance and regulate hepatic lipogenesis contributing to the development of liver steatosis.

## PKCs and PKDs regulate differentiation and function of adipocytes

Mammalian adipose tissue possesses a remarkable capacity to expand and to adapt to the fluctuations in nutrients supply. Thus, adipocytes have a major role in preventing the ectopic accumulation of fat in organs such as liver, skeletal muscle, heart, and pancreas [80]. Adipose tissue regulates energy storage, adaptive thermogenesis, endocrine function, food intake and provides fuels to peripheral organs. Adipocytes are classified into three types, based on their main features; white, beige, and brown adipocytes. Of note, beige adipocytes can emerge from white adipocytes or adipogenic precursors [81, 82]. Importantly, all of adipocytes types can store fat and produce adipokines, however, only beige and brown adipocytes can dissipate energy in the form of heat. For this reason, targeting beige and brown adipocytes represents a promising strategy to counteract obesity and type 2 diabetes.

Classical reports from the early 90s proposed a possible link between insulin signaling and PKCs in adipocytes and identified PKCβ, PKCγ and PKCε as the main PKC isoforms in adipocytes [83, 84]. Over the last two decades, significant advancements were made in the field of PKCs and their relevance in adipose tissue biology and obesity [85]. Importantly, the inactivation of PKCγ and PKCε impairs adipose differentiation in 3 T3-L1 cells by affecting the expression of PPARγ and CEBPα, essential adipogenic markers, while other PKC isoforms do not influence differentiation process [86]. However, other PKCs might influence other aspects of adipocytes biology. For instance, PKCβ deletion in the whole body of mice protects against diet-induced obesity. These animals present reduced adiposity, higher energy expenditure, increased expression of oxidative genes, improved mitochondrial fitness, higher levels of adrenergic receptors to sustain fat mobilization [87, 88]. Consistently, rodent models of obesity present higher levels of PKCβ in adipose tissue [87]. In line with this, human data have shown that activation of PKCβ by antipsychotic drugs promotes adiposity [89] and single nucleotide polymorphisms in the PKCβ promoter correlate negatively with insulin sensitivity [90]. Deletion of another isoform, PKCε, in adipose tissue results in protection from diet-induced insulin resistance and glucose intolerance [55]. Moreover, PKCε might promote glucose uptake in adipocytes while PKCθ inhibits adiponectin expression [91–95].

PKDs in adipocytes are activated by DAGs and extracellular purines [77]. Recently, it was demonstrated that PKD1 deletion in adipocytes protects against obesity and diabetes, by inducing thermogenic adipocytes (beige cells) and promoting the expression of genes activating energy dissipation by adipocytes such as uncoupling protein 1 (UCP1), PR-domain containing 16 (PRDM16) or peroxisome proliferator-activated receptor gamma coactivator 1α (PGC1α) [79]. Moreover, the deletion of PKD1 in adipocytes promotes mitochondrial fragmentation and biogenesis. Finally, it was shown that PKD1 regulates adipocyte’s function by targeting and suppressing the activity of AMP-activated protein kinase (AMPK), as all the phenotypes observed in mice deficient for PKD1 in adipocytes were reversed by AMPK inactivation [79]. However, the impact of other PKD isoforms, PKD2 and PKD3 on adipose tissue function needs to be investigated in future.

Therefore, PKCs and PKDs regulate adipocytes’ acquisition and multiple aspects of their function.

## PKCs and PKDs regulate insulin secretion in pancreatic β-cells

The main function of pancreatic β-cells found in the endocrine part of pancreas – pancreatic islets, is to produce and release insulin in response to the elevated glucose level in blood. Transport of glucose into β-cells is followed by the closing of the ATP-sensitive potassium channel and depolarization that leads to calcium influx via voltage-gated calcium channel [96]. Subsequently, the calcium influx activates PLC, which in turn hydrolases PIP_2_ and generates DAG [97]. Moreover, PLC in β-cells can also be activated through G-protein-coupled receptor pathways [98, 99]. An elevated level of DAG in β-cells causes activation of PKCs and PKDs and is associated with increased insulin secretion [96]. However, prolonged accumulation of intracellular lipids can be toxic, may promote β-cell failure, and contribute to type 2 diabetes development [100]. Moreover, sustained fatty acid overload results in the synthesis and accumulation of DAG in β-cells, which is correlated with impaired insulin secretion [101].

Several studies proved that treatment with an unspecific PKC activator, phorbol 12-myristate 13-acetate (PMA), stimulates insulin secretion in β-cells [102, 103]. PMA does not mediate intracellular calcium influx, nevertheless, it potentiates sensitivity of β-cells to calcium, decreasing the concentration of calcium needed to promote insulin exocytosis [103]. The effect of PMA was first linked to activation of PKCs, nevertheless, this agonist was also shown to activate PKDs [104]. PKCα, PKCβI, PKCβII, PKCδ, PKCθ, PKCη, and PKCε are expressed in pancreatic β-cells [105, 106]. Several studies showed that PKCs inhibitors strongly attenuate insulin secretion. Ro 31-8220, a non-selective PKC inhibitor partially reduced glucose-induced insulin secretion [107]. Moreover, Gö6976, an inhibitor of cPKCs significantly reduced the second phase of glucose-induced insulin secretion but not the initial one [108]. It was also observed that alike PMA, glucose and potassium-driven depolarization can cause translocation of PKCs to the plasma membrane [109–114]. Translocation dynamic upon DAG elevation in the plasma membrane differs depending on the isoform of PKC [105]. Interestingly, Yedovitzky et al. showed that glucose stimulation induces translocation of PKCα and PKCε to the periphery but PKCδ to the perinuclear site [110]. Furthermore, inhibition of PKCα and PKCε translocation reduces glucose-induced insulin release [110]. β-cells treatment with calcium-free buffer results in decreased translocation of PKCα, but not PKCε, and is associated with partially abolished insulin response [110]. Several studies showed the importance of PKCα in glucose and potassium-induced insulin granules secretion. However, Mendez et al. showed that after glucose treatment PKCε, but not PKCα, associates with insulin granules, which is essential for insulin exocytosis [112].

Overall, increased PKCs activity potentiates glucose-induced insulin secretion enhancing the second phase of insulin granule exocytosis. Based on the data obtained also from other cell types, it was proposed that PKCs regulate cortical actin rearrangement that facilitates insulin secretion and that the effect of PKCs is dependent on such exocytotic proteins as synaptotagmin, the mammalian homolog of UNC-18 (Munc18) and synaptosomal nerve-associated protein 25 (SNAP-25) [96]. However, the exact molecular mechanism of PKCs action on insulin release process in β-cells is not fully known yet and needs further studies. Pathways potentially involved in this phenomenon are discussed in detail elsewhere [96].

Additionally, PKCs are also involved in non-glucose-stimulated insulin release in β-cells. PKCs are shown to be effectors of glucagon-like peptide 1 (GLP-1) [115]. Shigeto et al. showed that GLP-1 activates PLC, which in turn leads to PKCs activation and subsequently membrane depolarization and insulin release [115]. The PKCs-mediated effect of GLP-1 is dependent on the activation of Na^+^-permeable transient receptor potential ion channels (TRPM) 4 and 5 [116]. It was also observed that PKCs inhibitors partially attenuate fatty-acid-induced enhancement of insulin secretion [117–119]. Moreover, using several inhibitors it was shown that nPKCs mediate the signal from non-nutrient secretagogues and enhance mitochondrial respiration and subsequently potentiate insulin release in INS1E cells and human pancreatic β-cells [120].

Several studies showed that fatty acid-treatment increases PKCs activity in β-cells [121–123]. Since elevated lipid level is known to induce β-cell dysfunction, PKCs were linked to the development of metabolic disorders [124]. Mice with global deletion of PKCε present enhanced insulin secretion upon fatty acids treatment and improved glucose tolerance when fed with HFD [124]. Genetic and functional ablation of PKCε results in an increased insulin secretion ex vivo in islets derived from diabetic mice deficient for the function of leptin receptor (*db/db* mice) which were pretreated with lipids. It was also observed that PKCɛ deletion restores the balance between lipid esterification and oxidation altered by fatty acid treatment in pancreatic islets [124]. Moreover, using PKCɛ-inhibitory peptide improves glucose-induced insulin release and glucose tolerance in diabetic *db/db* mice [124]. Similarly, overexpression of kinase-negative PKCδ in mice protects from HFD-induced glucose intolerance, increases insulin level and pancreatic islets size, and decreases apoptosis marker cleaved caspase-3 in β-cells in comparison to control animals [125]. Furthermore, overexpression of kinase-negative PKCδ protects isolated islets and INS1E cells from palmitic acid-induced apoptosis and mitochondrial dysfunction [125]. However, β-cell-specific overexpression of wild type PKCδ does not influence glucose tolerance, insulin plasma level or islet size in mice fed both, standard laboratory diet and HFD [126]. Moreover, insulin content and glucose-induced insulin secretion in INS1E cells overexpressing wild type PKCδ were similar to control, which shows that increased level of PKCδ did not impair β-cell function [126]. This data indicate that PKCδ and PKCε are important players in lipid-induced β-cell dysfunction and pathogenesis of type 2 diabetes, however PKCδ upregulation in β-cells is not sufficient to induce diabetic phenotype.

Streptozotocin treatment induces type 1 diabetes and this model is widely used to study autoimmune distruction of β-cells [127]. Global deletion of PKCδ delays the onset of hyperglycemia in streptozotocin-treated mice, confirming the importance of this PKC isoform in the pathogenesis of type 1 diabetes [128]. Furthermore, it was shown that the deletion of PKCδ in pancreatic islets protects from cytokine-induced apoptosis, NO generation, and also disrupts toll-like receptor 2 (TLR2) signaling, which activation contributes to the development of type 1 diabetes [128–130]. GLP-1 analog exendin-4 can suppress apoptosis and protects against oxidative damage in β-cells. By using specific inhibitor Kim et al. showed that the effect of exendin-4 on the expression of antioxidant genes mediated by nuclear factor erythroid 2-related factor 2 (Nrf2) in β-cells upon stress is dependent on PKCδ activity [130]. Apoptosis in β-cells is also regulated via transcription factor forkhead box protein O1 (FOXO1), that in dephosphorylated form increases expression of pro-apoptotic genes. It was shown that PKCδ regulates nuclear retention of FOXO1 in β-cells via phosphorylation of 14–3-3ζ chaperone [126]. Under non-stress conditions, both, INS1 overexpressing PKCδ and islets derived from mice with β cell-specific overexpression of PKCδ are characterized by increased accumulation of phosphorylated form of FOXO1 in the nucleus but do not display increased apoptosis [126]. Taken together, these data indicate an important role of PKCδ in the regulation of β-cell death and progression of type 1 diabetes development.

All the PKD isoforms are expressed in pancreatic islets, nevertheless, PKD1 is a dominant isoform [131–133]. PKD1 promotes granule secretion and fission of vesicles destined for exocytosis from the trans-Golgi network (TGN) [134]. It was shown that PKD1 plays an important role in the regulation of insulin secretion and the survival of β-cells in the pathogenesis of diabetes mellitus [132]. In β-cells, PKD1 activity is regulated at multiple levels. PKD1 is activated by the neurotransmitter derived from the parasympathetic nervous system, acetylcholine, and inhibited by mitogen-activated protein kinase (MAPK) p38δ (which directly phosphorylates PKD1) upon oxidative stress. Consistently, deletion of p38δ in mice leads to elevated PKD1 activity, enhanced insulin granule secretion, better glucose tolerance and protects against pancreatic β-cell failure [132]. Moreover, PKD1 is also a downstream effector of fatty acid receptor 1 (GPR40), which promotes the second phase of fatty acid-induced insulin release, and controls actin cytoskeleton remodeling in β-cells [135]. On the molecular level, PKD1 controls the biogenesis of insulin granules at the TGN by phosphorylating a BAR-domain-containing protein Arfaptin-1 at serine 132 [136]. Arfaptin-1 prevents the premature fission of the secretory granules from the TGN. PKD-dependent phosphorylation of Aprfaptin-1 allows fission of the insulin granules TGN and consequently its secretion [136].

Recently, PKDs were found to mediate adaptation to nutrient availability in β-cells. Upon feeding, PKDs block starvation-induced nascent granule degradation (SINGD), therefore promoting insulin granule secretion. De-activation of PKDs during nutrient deprivation allows the fusion of insulin granules with lysosomes and their subsequent degradation. This leads to the activation of the mechanistic target of rapamycin (mTOR) and subsequently autophagy suppression, which is required to inhibit insulin secretion during nutrient deprivation [137]. Another study showed that PKD prevents SINGD, partially in the CD63-dependent manner. Inhibition of PKD induces SINGD, which contributes to the β-cells failure in diabetic BTBR^ob/ob^ mice [131]. Mice carrying β-cell-specific inducible deletion of PKD1 present similar glucose tolerance and insulin levels as control animals when fed a standard diet. However, when fed with HFD mice with PKD1 deletion in β-cells were characterized by glucose intolerance and impaired glucose-induced insulin release [138]. This suggests that PKD1 is involved in β-cell adaptation to increased insulin demand upon HFD and protects against β-cell dysfunction [138].

Xiao et al. for over 3 years studied a cohort of rhesus monkeys with spontaneous metabolic syndrome. They observed that monkeys with hyperinsulinemia, which is a condition implicated in the pathogenesis of insulin resistance and type 2 diabetes, were characterized by highly reduced expression of PKD2 due to nonsense mutation (K410X) [133]. It was also shown that at a young age global PKD2 knockout mice are characterized by increased fasting insulin level and decreased fasting glucose level in comparison to control animals [133]. Moreover, adult mice with PKD2 deficiency exhibit increased basal and glucose-stimulated insulin secretion, which is associated with augmented L-type calcium channel-dependent calcium influx in response to membrane depolarization induced by potassium chloride and glucose in those animals. Adult PKD2 knockout mice are also characterized by improved glucose tolerance and slightly decreased insulin tolerance [133].

Taken together, PKCs and PKDs play an important role in the regulation of insulin secretion and the survival of pancreatic β-cells.

## PKCs suppress insulin sensitivity in skeletal muscle while PKDs are required for their function

Skeletal muscle is an organ that accounts for approximately 45% of the human body and is mainly responsible for body movement [139]. Since skeletal muscle is a highly metabolically active tissue responsible for up to 90% of glucose disposal in the postprandial state, it is considered to play a crucial role in maintaining whole-body energy homeostasis and development of metabolic disorders, for instance, insulin resistance and glucose intolerance [140]. Increased accumulation of DAG is inversely correlated with insulin sensitivity and is one of the factors causing the development of insulin resistance in skeletal muscle [141]. Moreover, elevated DAG level in muscles is associated with activation of PKCs and PKDs [142–145].

PKCα, PKCβ, PKCδ, PKCθ, and PKCε are expressed in skeletal muscle. However, PKCθ is recognized as a dominant isoform of DAG-sensing PKCs in this tissue [146]. Several studies have shown profoundly altered expression levels and activity of PKCs in skeletal muscle of obese human patients and rodent models of diabetes in comparison to healthy and lean controls [142, 143, 147–149]. In 1997, Bandyopadhyay et al. showed that PKCζ, but not DAG-dependent PKCs, is involved in insulin-stimulated glucose uptake in L6 myotubes in PI3K dependent manner [150]. However, Itani et al. observed increased translocation of PKCβ, PKCδ, and PKCθ from the cytosol to the membrane after insulin treatment in human skeletal muscle indicating its increased activation [148]. Moreover, insulin treatment causes alternative splicing of PKCβ pre-mRNA in favor of the PKCβII isoform and its activation is mediated by PI3K in skeletal muscle cells. In turn, activated PKCβII promotes glucose transportation via phosphorylation of its substrate myristoylated alanine-rich C-kinase substrate (MARCKS) [151].

Lack of PKCθ protects skeletal muscle from lipid-induced insulin resistance [152]. HFD-fed mice with skeletal muscle-specific PKCθ deficiency are characterized by decreased intracellular lipid accumulation and increased insulin sensitivity in skeletal muscle, decreased fasting glucose level, and lower daily calorie intake followed by lower weight gain in comparison to control mice [153]. Moreover, it was shown that activation of PKCθ induces inhibitory phosphorylation of IRSs at several residues, e.g. pSer1101 and pSer307, which is associated with disruption of insulin signaling pathway and decreased PI3K and AKT activity [154, 155]. Global deletion of PKCα increases insulin-stimulated glucose transport in skeletal muscle in mice whereas knockout of PKCβ increases both basal and insulin-stimulated glucose uptake in isolated soleus muscle [156, 157]. PKCε is also implicated in regulating skeletal muscle metabolism. Administration of PKCε abrogating peptides protect skeletal muscle from diet-induced insulin resistance and decreases phosphorylation of IRS-1 [158]. Interestingly, skeletal muscle-specific knockout of PKCδ improves age-related decline in whole-body insulin sensitivity and glucose tolerance, and also increases insulin sensitivity of skeletal muscle in older mice but does not protect from HFD-induced insulin resistance. Moreover, skeletal muscle with deficiency of PKCδ is characterized by decreased metabolic rate and lower level of OXPHOS proteins, indicating an important role of PKCδ in the regulation of mitochondria homeostasis at an older age [159]. The data published up to date indicate that all studied DAG-sensing PKCs regulate insulin pathways by providing a negative feedback loop, subsequently decreasing insulin-stimulated glucose uptake and other insulin-dependent processes in muscle cells. Overall, PKCs play a crucial role in DAG-induced insulin resistance in skeletal muscle.

Exercise is found as an effective treatment of insulin resistance in peripheral tissues. Rao et al. showed that physical training decreases the level of PKCβ but not of other PKC isoforms in skeletal muscle of HFD-fed mice. Knockout of PKCβ protects mice from HFD-induced insulin resistance in skeletal muscle in a similar way to exercise. Moreover, physical training does not influence skeletal muscle insulin sensitivity in PKCβ deficient mice fed with HFD. This data suggests that exercise protects against HFD-induced insulin resistance in skeletal muscle at least partially through the downregulation of PKCβ [160].

Moreover, it was shown that PKCθ regulates myoblast differentiation in C2C12 cells. PKCθ knockdown reduced inhibitory IRS-1 phosphorylation, increased extracellular signal-regulated kinase (ERK) 1/2 phosphorylation, enhanced myoblast differentiation and cell fusion, and increased protein synthesis [161]. Also, PKCε in skeletal muscle is involved in the differentiation of muscle cells and myofiber regeneration. During differentiation, PKCε localizes in the nucleus and blocks Hmga1 gene expression to promote myoblast formation [162].

In skeletal muscle PKCε and PKCβ1 are also involved in transmitter release at the neuromuscular junction [163, 164]. It was shown that both PKCε and PKCβ1 are localized in the motor nerve terminals of the neuromuscular junction. Expression and phosphorylation level of those PKCs in the synaptic membrane is increased in response to electrical stimulation and muscle contraction via the brain-derived neurotrophic factor (BDNF)-mediated tyrosine kinase receptor B (TrkB) signaling [163, 164]. Additionally, it was shown that muscle contraction regulates PKCβ1 phosphorylation in the synaptic membrane through phosphoinositide-dependent kinase 1 (PDK1) [164]. PKCε causes phosphorylation of MARCKS involved in neurotransmission-related actin cytoskeleton remodeling [163]. Using a specific inhibitor it was also shown that PKCε is involved in the regulation of acetylcholine release in the neuromuscular junction [163].

PKCs are involved in saturated fatty acid-induced proinflammatory signaling in skeletal muscle. Palmitic acid treatment increases intracellular DAG and subsequently induces PKC-dependent Ser153 phosphorylation of Raf-kinase inhibitor protein (RKIP) that results in RKIP-Raf1 dissociation and activation of ERK signaling pathway [165]. It is known that inflammation plays an important role in the pathogenesis and progression of skeletal muscle disorders, for instance, insulin resistance and Duchenne Muscular Dystrophy (DMD). DMD is a severe muscle disease caused by a mutation in the dystrophin gene. It was shown that knockout of PKCθ in the mouse model of DMD (mdx) prevents muscle inflammation, reduces muscle wasting, improves muscle regeneration and maintenance of performance [166]. Administration of PKCθ specific inhibitor to young mdx mice also leads to a reduction in muscle damage, immune cell infiltration, muscle inflammation, and maintenance of muscle regeneration [167]. These data suggest that specific PKCθ inhibitor may potentially be used in the treatment of DMD [167].

All of the PKD isoforms are expressed in skeletal muscle; however, the level of expression can differ between myofiber types [168, 169]. For instance, PKD1 is the most abundant isoform and is predominantly expressed in type I oxidative, slow-twitch myofibers. Moreover, it was shown that overexpression of constitutively active PKD1 in type II glycolytic, fast-twitch myofibers leads to transformation into type I myofibers phenotype [169]. Skeletal muscle consisted mostly of type I myofibers in mice with skeletal muscle-specific PKD1 knockout and are characterized by increased susceptibility to fatigue [169]. The data suggest that PKD1 regulates muscle fiber phenotype through phosphorylation of class II histone deacetylases (HDACs), a repressors of myocyte enhancer factor-2 (MEF2) transcription factor regulating the expression of genes involved in differentiation of myoblasts into multi-nucleated myotubes [169–171]. Moreover, it was shown that PKD1 mediates HDAC5 nuclear efflux as an effector of α-adrenergic signal, which is typically present in parallel with motor neuron input during physical training [172]. It was also shown that mice with overexpression of dominant-negative PKD1 exhibited decreases running performance and significantly impaired voluntary running-induced myofiber transformation in skeletal muscle, which confirms that PKD1 plays a crucial role in skeletal muscle remodeling upon physical training [173]. Coughlan et al. showed that PMA acting as DAG mimetic increases phosphorylation of AMPK, one of the major energy regulators in the cell, decreasing activity of its subunit AMPKα2 in the time and dose-dependent manner in C2C12 [144]. PKD1 specific inhibitor and PKD1 knockdown fully prevent PMA-induced effects on AMPK, whereas PKCs inhibitor acts only partially. Interestingly it was also shown that PKD1 inhibitor protected C2C12 cells from insulin resistance induced by PMA [144]. Moreover, PKD1 was found to directly phosphorylate AMPKα2 at Ser491 in in vitro studies [144]. These data suggest that PKD1 can be involved in insulin signaling impairment via AMPK inhibition.

Inhibition of PKDs decreases myotube fusion and myoblast differentiation in primary mouse satellite cells and C2C12 stable cell line [168]. Furthermore, it was shown that PKD2 is more relevant in the regulation of those processes than PKD1 and PKD3 isoforms. PKD2 is phosphorylated and, at the same time, activated during the initiation of C2C12 myoblasts differentiation, whereas knockdown of PKD2 leads to inhibition of myoblast cell fusion and impaired expression of muscle development-associated genes [168]. Kleger et al. observed that PKD2 activates the promyogenic transcription factor MEF2D via inhibition of paired box gene 3 (Pax3) [168]. Overexpression of dominant-negative PKD3 inhibits basal glucose transport but has only a minor effect on insulin-stimulated glucose uptake. Additionally, overexpression or silencing of PKD3 causes respectively, significant increase or decrease in basal glucose uptake in L6 myotubes [174]. Moreover, using a truncated form of the PKD catalytic domain, it was shown that PKDs also regulate expression of proteins involved in glucose and lipid metabolism, β-oxidation, and OXPHOS in C2C12 myotubes both in HDAC5-dependent and independent manner [175].

In conclusion, PKCs in skeletal muscle suppress insulin action while PKDs promotes muscle differentiation and function.

## PKCs and PKDs in the regulation of appetite and food digestion

The regulation of food intake and nutrients absorption in the gastrointestinal tract determines glucose and lipid homeostasis. The hypothalamic arcuate nucleus plays a central role in the integration of hormonal and nutritional signals which regulate metabolic homeostasis [176]. Animals fed HFD present increased intracellular DAG levels in the hypothalamus [177]. Nevertheless, the impact of PKCs and PKDs on the hypothalamic regulation of metabolism is poorly understood. Administration of specific PKC agonists into the hypothalamus suppresses hepatic gluconeogenesis. Importantly, this effect can be reversed by PKCδ-specific inhibitor, rottlerin [178]. On the other hand, palmitic acid promotes the hypothalamic accumulation of DAG to stimulate the translocation of PKCθ to the plasma membrane. Importantly, PKCθ mediates palmitic acid-induced suppression of insulin signaling in the arcuate nucleus promoting body weight gain and glucose intolerance in mice fed HFD [179].

The gastrointestinal tract is the first side challenged by high-fat-containing foods. Therefore, it would be logical to assume that the fat challenge leads to the accumulation of DAG and subsequent activation of PKCs and PKDs in the intestine. However, up to date, there are no studies available that would cover this topic. Nevertheless, several studies implicated PKC and PKD isoforms in the regulation of intestinal epithelial cells proliferation and differentiation [180, 181].

## PKCs and PKDs – beyond the regulation of glucose and lipid homeostasis

DAG evoked activation of PKCs and PKDs were also broadly discussed in the context of heart function. However, these aspects were recently revised in [182, 183]. Similarly, PKCs regulate multiple aspects of innate [184] and adaptive immunity [185], while PKDs plays a central role in the regulation of immune response [186, 187]. However, these aspects, likewise the PKCs and PKDs impact on other biological processes are beyond the scope of this review.

## Targeting PKCs and PKDs – potential clinical applications

The PKCs and PKDs families play a pivotal role in metabolic regulation. Thus, targeting these kinases might represent a promising avenue for the treatment of metabolic disorders such as obesity and diabetes. Hitherto, several clinical trials have been carried out to target PKCs in the context of different clinical conditions. For metabolic research trials include diabetic retinopathy, congestive heart failure, coronary bypass grafting and acute myocardial infarction salvage [188–196]. Unfortunately, to date, due to limitations related to off-target effects, lack of specificity, lack of suitable metabolites in blood or urine to monitor the activity of the PKC inhibitors, as well as due to inconclusive preclinical studies, there is no commercially available drug on the market that targets specifically any of the isoforms of the PKCs [197]. Nevertheless, the pleiotropic effects of the PKCs make this family of kinases a potential target for the treatment of multiple metabolic diseases.

Concerning the PKD family there is no ongoing clinical trial register in clinicaltrial.gov. However, direct or indirect targeting of PKD isoforms might be an attractive strategy for preventing pancreatic β-cell failure during the onset of diabetes or for the treatment of obesity.

## Conclusion and future perspective

In summary, this review provides comprehensive and updated insights into the classification, structure, tissue distribution and functions of the DAG-sensing PKCs and PKDs in health and metabolic diseases, with a major focus on organs involved in metabolic regulation, such as liver, adipose tissue, pancreas, and skeletal muscle. Besides, this review provides a brief overview of the current state of the art in PKCs and PKDs drug discovery and development. DAG-sensitive PKCs and PKDs play a crucial role in the regulation of metabolism in peripheral tissues. However, understanding of the complex interplay between different DAG-sensitive kinases and other components of signaling machinery requires further investigation. Moreover, targeting specific members of PKC and PKD families might be beneficial for the treatment of metabolic diseases especially type 2 diabetes and obesity. Nevertheless, highly specific inhibitors of selected PKCs and PKDs would be required to ameliorate the potential side effects of therapies against obesity, diabetes, and associated diseases.

## Data Availability

Not applicable.

## Abbreviations

AMPK: AMP-activated protein kinase
aPKC: Atypical PKC
ATGL: Adipose triglyceride lipase
cPKC: Conventional PKC
DAG: Diacylglycerol
DAGK: Diacylglycerol kinase
DGAT2: Diglyceride acyltransferase
DMD: Duchenne muscular dystrophy
ER: Endoplasmic reticulum
ERK: Extracellular signal-regulated kinase
FOXO1: Forkhead box protein O1
GLP-1: Glucagon-like peptide 1
HDACs: Class II histone deacetylases
HFD: High-fat diet
HSL: Hormone-sensitive lipase
IRS: Insulin receptor substrate
MARCKS: Myristoylated alanine-rich C-kinase substrate
MCD: Choline-deficient diet
Mdx: Mouse model of Duchenne muscular dystrophy
MEF2: Myocyte enhancer factor-2
NAFLD: Non-alcoholic fatty liver disease
nPKC: Novel PKC
PA: Phosphatidic acid
PIP_2_: Phosphatidylinositol 4,5-bisphosphate
PKC: Protein kinase C
PKD: Protein kinase D
PL: Phospholipid
PLC: Phospholipase C
PMA: Phorbol 12-myristate 13-acetate
RKIP: Raf-kinase inhibitor protein
SINGD: Starvation-induced nascent granule degradation
TAG: Triacylglycerol
